# An Ultra-Thin, Triple-Band, Incident Angle-Insensitive Perfect Metamaterial Absorber

**DOI:** 10.3390/ma16041623

**Published:** 2023-02-15

**Authors:** MST Ishrat Jahan, Mohammad Rashed Iqbal Faruque, Md Bellal Hossain, Sabirin Abdullah

**Affiliations:** Space Science Centre (ANGKASA), Institute of Climate Change (IPI), Universiti Kebangsaan Malaysia, Bangi 43600, Malaysia

**Keywords:** metamaterial absorber, incident angle insensitivity, RAB

## Abstract

We created an ultra-thin, triple-band incident angle-insensitive perfect metamaterial absorber (MMA) with a metallic patch and a continuous metal ground isolated by a central dielectric substrate. The top metallic patch, placed across the edges of the 0.58 mm thickness Rogers RO4003C (lossy) substrate, forms the bulk of the projected absorber’s ultra-thin layer. Nonetheless, absorption is exceedingly strong, covering C-band, X-band and K-band and reaching levels of 97.8%, 99.9%, and 99.9%, respectively, under normal and even oblique (0° to 45°) incident conditions. In chosen ranges of frequency of 6.24, 10.608, and 18.624 GHz for both TM and TE mode, the displayed Q-factors were 62.4, 17.68, and 26.61, respectively. We correspondingly calculated the RAB (relative absorption bandwidth) to evaluate absorption performance. An equivalent circuit proved its performance capabilities, indicating that it would produce a high-quality MMA from ADS software. Furthermore, the absorber’s performance has been verified in free space on a sample being tested using a different array of unit cells. Moreover, the proposed structures with HFSS simulators to display the MMA’s absolute absorption at each absorption peak are somewhat inconsistent with the results of the CST simulator. Because of its superior performance, the ultra-thin absorber is suited for a wide range of applications, including satellite applications such as radar systems, stealth technology, imaging, and electromagnetic interference reduction.

## 1. Introduction

Metamaterials are artificially scheduled array formations exhibiting sub-wavelength dimensions. Their numerous spectacular electromagnetic characteristics make them potentially helpful for the study of nano-photonics [1,2]. Unusual reactions are brought about by electromagnetic waves interacting with metamaterials (MMs). Metamaterials can attain the performance of anisotropic epsilon-near-zero (ENZ) materials under specific situations by resolving Maxwell’s electromagnetic field formula [3,4,5]. Metamaterials have received a significant amount of attention owing to their exceptional electromagnetic properties, which are not found in nature, as well as their compact size and low loss performances [6]. The electromagnetic characteristics of these MMs can be characterized utilizing effective parameters such as effective permeability and effective permittivity [7,8], as well as the negative refracted index [9], antenna [10], super lensing [11], and cloaking devices [12]. According to the various application demands, metamaterial absorbers may generally be divided into two types: narrowband and broadband [13]. In solar energy harvesting and thermal emissions, broadband absorbers are widely employed. Because narrowband absorbers have benefits over conventional types, they are used for optical detection and sensing applications such as customizable miniaturization, improved flexibility, and greater efficiency. The effective impedance of a metamaterial absorber may be tuned so that absorption is close to perfect at a specific frequency [14].

Many scholars in the electromagnetic science community have examined the electromagnetic behaviors of MMs in order to demonstrate their broad usefulness in the creation of efficient devices given their exotic electromagnetic properties [15]. In addition to being polarization-insensitive and omnidirectional, the perfect MMA should have an absorbing coefficient closer to unity [16]. In order to create metamaterial absorbers with excellent absorption capabilities, lossy materials are often arranged in periodical array patterns [17]. These configurations can efficiently contain and absorb incident electromagnetic (EM) waves [18]. Extensive use has been made of electromagnetic (EM) absorbers for both civilian and military uses, including electromagnetic compatibility, electromagnetic shielding, and stealth technology [19]. Metamaterials are frequently employed to improve the operation of microwave devices [20].

MMs have concentrated on the real components of ɛ and µ, and normally, it is desirable to minimize the loss represented by imagined portions of ɛ and µ [21]. The ground plane, in combination with the periodical metal resonant structure, produces the magnetic response while tuning the magnetic permeability. The periodical metal resonant structure produces an electrical response to adjust the electric permittivity [20]. Double negative (DNG) materials were the subject of Veselago’s research in 1968, but new techniques have since been discovered in the microwave regimes [11]. Due to their exceptional qualities, DNG materials are widely recognized as metamaterials [12] while maintaining the thinnest thickness achievable [10]. A thin, ideal metamaterial absorber that simultaneously excites electric resonant frequencies to achieve the impedance bandwidth with surrounding atmosphere, thereby removing any edge reflection, was initially suggested by Landy et al. in 2008 [22]. Metamaterial absorbers developed quickly because they are less bulky, easier to fabricate, and lighter than conventional absorbers [23]. The designing of metamaterial absorbers with an appropriate working frequency spectrum, a decent wide-angle tolerance, bandwidth, polarization, a compact size, insensitivity, and a simple fabrication method, however, is difficult in practice. An ideal metamaterial absorber often has a small bandwidth due to its resonance characteristics [24]. Consequently, much work has been carried out to increase the bandwidth of metamaterial absorbers so that they may be used in more real-world applications [25]. These absorption-frequency properties of absorbers can be used to assess their behavior. Typically, conventional ideal absorbers have assumed the shape of copper ring resonators on a ground plane, and, thus, are intended to reduce transmissions and reflections while enhancing absorption [26]. The best technique to enhance the maximum absorption is to incorporate a variety of metallic resonators of various sizes into the crystalline structure. Different-sized resonators can generate absorption peaks at various particular frequencies, and their superposition effect will result in ideal absorption throughout several frequency ranges [27]. Researchers have presented many methods for increasing the quality factor of these sensors, including printing them onto low-loss dielectric substrates [14]. It is possible to tune for a high-quality factor depending on absorptions (the transition percentage and the reflection) [28]. Q = f/FWHM [29] can be employed to calculate the quality factor, where f is the resonance frequency and FWHM is the full width at half maximum. Given the configurations of the metamaterial absorbers discussed above, insensitivity to varied polarizations and incident oblique angles represents one of the proposed design considerations for MMA. The symmetrical geometry of the unit cell’s horizontal and vertical axes causes a condition known as polarization insensitivity [23]. The projected MMA’s excellent absorption rate, simplicity to manufacture, ultra-thinness, and light weight make it preferable to a traditional absorber.

It is important to clarify that the goal of our design is not to enhance a specific feature of the MA, but rather to maintain very high absorption at an incident angle of both TE and TM polarization in a particular medium range of frequencies. In this article, we present an ultra-thin incident angle-insensitive triple-band metamaterial absorber. When facing both TE and TM polarized waves, however, incident angle-insensitive structures consist of just a metallic patch, a dielectric layer, and a copper ground. The physical process of perfect absorption in several frequencies can be described using the theory of electric dipole resonance [12]. The novelty of this research is that the proposed ultra-thin metamaterial absorber produces triple absorption peaks of 97.8%, 99.9%, and 99.9% at 6.24 GHz, 10.608 GHz, and 18.624 GHz, respectively, covering C-band, X-band, and K-band. A better Q factor and relative absorption bandwidth can be provided. The double metallic symmetric split ring resonators (SRRs) with H-shaped structures can be presented in a unique shape, with compactness and ultra-thin characteristics. The metamaterial absorber shows nearly perfect absorptions over a wide angle of incidences, up to 45°, for both the TE and TM modes. Due to its better performance, the ultra-thin absorber is suited for a wide range of applications, such as satellite applications including radar systems, stealth technology, imaging, and electromagnetic interference reduction. In order to better understand the resonance process, surface current and electromagnetic field distributions are also analyzed herein. Importantly, we show in this study that the proposed absorber can maintain a high degree of absorbance at significant polarization angles.

## 2. Metamaterial Unit Cell Design Geometry

**Figure 1:**
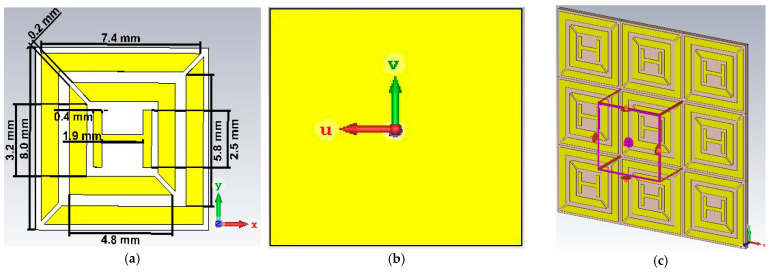
A proposed absorber of (**a**) dimension view; (**b**) back side view; (**c**) simulation view.

Square-shaped patch exclusion is the basis of the proposed design. This work proposes a downsized, incident angle-insensitive, ultra-thin metamaterial absorber that is developed using the resonant method. The unit cell size of the metamaterial absorber is 8 mm × 8 mm, comprising two metallic square patches with H-shaped structures that have been etched onto a Rogers (lossy) substrate to realize insensitive oblique incidence angle properties and three separate resonant frequencies, as shown in Figure 1a. The suggested metamaterial absorber is made up of three layers (sandwiched structure): a layer of grains serving as a unit cell layer at the top, a dielectric layer with an ultra-thin substrate in the middle, and a metallic ground plate at the bottom.

Models for the metallic resonator and ground plate include copper sheets [30] with electrical conductivities of σ = 5.8 × 10^7^ S/m and 0.035 mm in thickness. Because the thickness of the copper sheet utilized in this application is significantly greater than the average skin depth in the microwave frequency range, the only thing preventing the absorption is reflection. The back-side view is indicated in Figure 1b. We selected the Rogers RO4003C (lossy) dielectric substrate for the intermediate layer, as the key benefit of the Rogers 4003C is that it is a low-loss material, with good mechanical strength, two types of glass cloth, and a comparably low cost compared to other substrates. As a result, it possesses excellent electrical and mechanical characteristics, as well as a low Tg (thermal expansion coefficient). It has a thickness of h = 0.58 mm, loss tangent of tanδ = 0.0027, permeability of µ = 1, and permittivity of ε = 3.55. The metamaterial unit cell size is 8 mm × 8 mm. Here, the length and width of the substrate and ground layer are 8 mm in this case, the split gaps of both rings are 0.2 mm, the length and width of the outer ring are 7.5 mm and 5.9 mm, the length and width of the inner ring are 4.9 mm and 3.3 mm, and the length and width of the sub-tracing H-shaped patch are 2.5 mm and 1.9 mm. The distance between two adjacent, overlapping striplines is 1 mm, and the distance between the substrates and the outer ring is 0.5 mm.

Numerical tests using CST Microwave Studio^®^, which is built on the finite integration approach and uses the frequency-domain solver for design simulation, were carried out to demonstrate the performance of the designed absorbers. The unit cell can be seen as an infinite lattice structure which is implemented using unit cell boundary conditions in both the x and y dimensions, while leaving the z direction open, as indicated in Figure 1c. The x or y polarized Floquet terminal excitations serve as a source or detectors of electromagnetic wave energy. Microwave sources and structures are separated by 5 mm. Under the influence of polarization microwave propagation on the z axis, the frequency domain solver was used to perform the needed calculations and subsequently produce the desired results for the numerical model.

## 3. Results and Discussion

### 3.1. Absorptions Scenario

**Figure 2:**
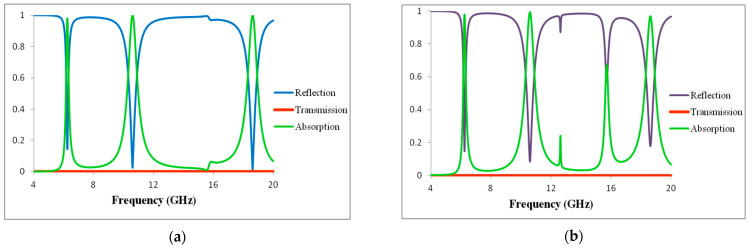
Absorption, reflection, and transmission results for (**a**) TE and (**b**) TM mode.

Figure 2a,b illustrate the metamaterial absorption, reflection, and transmission property of the TE and TM modes. The design presents triple bands with absorptivity rates of 97.8%, 99.9%, and 99.9% at 6.24 GHz, 10.608 GHz, 18.624 GHz, respectively, for TE mode. The proposed structure also exhibits triple bands with absorptivity levels of 97.8%, 99.3%, and 96.8% at frequencies of 6.24 GHz, 10.608 GHz, 18.624 GHz, respectively, for TM mode. The TM mode also shows an infant peak of 67% at 15.712 GHz. The FWHM values of the TE mode are 0.1, 0.6, and 0.7 GHz at resonance frequencies. The FWHM values of the TM mode are 0.1, 0.6, and 0.6 GHz. The higher and lower frequency bands correspond to the absorption bands with absorption levels of 97.8%, 99.9%, and 99.9% at 6.24 GHz, 10.608 GHz, and 18.624 GHz. The quality factor value of the specific structure allowed for a direct evaluation of its sensing performance. The quality factor is often defined as the resonant frequency point divided by the FWHM value, and it serves as an important indicator when determining whether to utilize the resonance mode. When the quality factor value is larger, the sensing performance is improved. The metamaterial absorber (MMA) has a strong absorption performance, with RABs up to 32%, 28%, and 21% for the TE and TM modes. For the TE and TM modes, Table 1 and Table 2 provide a summary of the quality factor (Q), full-width at half-maximum (FWHM), and relative absorption bandwidth resonance frequencies.

**Figure 3:**
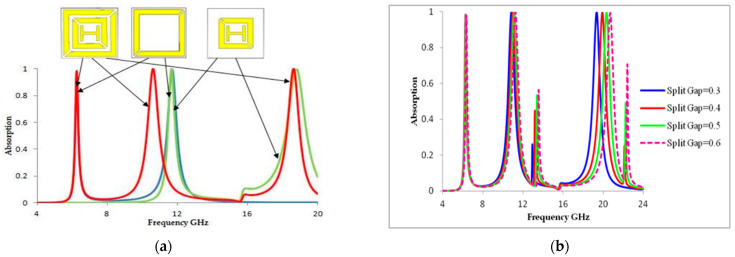
Absorption response at (**a**) different design steps (Red color mentioned in 1st model, Blue color mentioned in 2nd model, and yellow color mentioned in 3rd model); (**b**) different split gaps of the square ring.

Figure 3a illustrates that the different bands created as proposed unit cells indicate different parts of various stages. Creating a first and second absorptions peak of 97.8% and 99.9% at resonant frequencies 6.24 GHz and 12.16 GHz is made possible by the outer split ring resonator. The inner split ring resonator and H-shaped structure presents shifted second and third absorption peaks of 99.9% and 99.9% at resonant frequencies 12.16 GHz and 18.624 GHz. The outer split ring, inner split ring resonator, and H-shaped structure create a common absorption peak of 99.9% at a resonant frequency of 12.16 GHz. We noted that the suggested MMA should display band creation, as the designed unit cell consists of various steps at various parts of the process, but each part is very important for creating the resonance band. Each component of the structure determines its own resonance frequency, as seen in Figure 3a. As a result, combining the components of the entire structure results in a perfect triple-band metamaterial absorber. Consequently, we can attain the best simulated absorbance: A (ω) = 1 – T (ω) – R (ω) is little below unity A (ω) = 99% [31,32,33,34,35]. We observed high frequency reflectivity and produced values close to 99%. In Figure 2a, a great feature occurring in R (ω) is shown, with a minimum of 0% at a resonant frequency of 18.624 GHz. T (ω) passes through zero at all resonant frequencies. Therefore, the highest simulated absorbance of our MM unit cell is located close to where R (ω) and T(ω) have their minimums. In [36], the impedance and refractive index were directly connected to both permeability µ (ω) and permittivity ε (ω):μ (ω) = nz, ε (ω) = n/z. The metamaterial absorber fulfilled the requirement of this equation [37,38]: $\sqrt{\frac{\mu \;\left(\omega \right)}{\varepsilon \;\left(\omega \right)}}$ = Z (ω) = 1. In order to explain the absorption capability of MA, RAB (relative absorption bandwidth) was employed. The mathematical equation followed [21,25]: RAB = 2 × $\frac{f_{U-}f_{L}}{f_{U+}f_{L}}$. Here, f_L_ and f_U_ are the lower and upper limits of a frequency range with A (ω).

We can observe that the resonance frequency of the proposed unit cell depends on the split gap of the both inner and outer split ring, as well as the length of the copper. Figure 3b illustrates the absorption scenario of the proposed MA when the split gap of both square patches changes. An iterative approach was used to test several configurations until the required characteristic was attained. Although they varied very little, the resonance frequencies were constantly linear above 97.8%, 99.9%, and 99.9%. When the gap is 0.4, 0.5, or 0.6, two new bands are created at 13.2 GHz and 22.4 GHz. The implications of varying the split gaps of both the inner and outer ring of the proposed metamaterial unit cell were also explored. Sequential increments of gaps 0.3 to 0.6 decreased the capacitance, but increased the mutual inductance, because the resonance frequencies increased with the metal layer, as did the two split rings. As a result, we can observe that the capacitance value decreased as the split gap of the both the inner and outer rings rose, causing the resonant frequency to shift to the right. This method helps parametric studies to enable us to select the ideal resonator diameter.

### 3.2. Incident Angle Insensitivity

**Figure 4:**
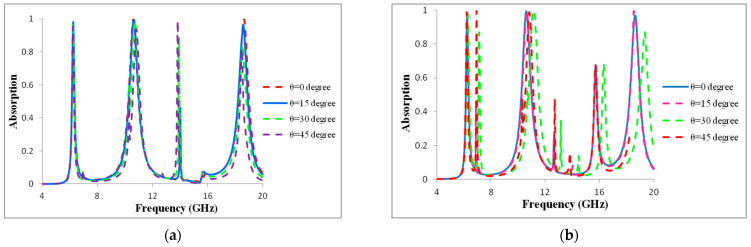
Absorption rate of different incidence angle (**a**) TE mode and (**b**) TM mode.

The metamaterial absorber must consistently absorb radiation at various incidence angles in order to be used in practical applications. We analytically investigated the metamaterial absorber’s angle dependency. The numerically observed absorptions are shown in Figure 4a,b under both the TE and TM modes. The angle of incidence varied from 0° to 45° over the entire relevant frequency spectrum (4 to 20 GHz). While varying the angle values, the structure’s absorption rates and resonance frequency remained constant. At every resonance, the proposed absorber displayed magnetic and electric dipole moments. The performances unavoidably suffered because the proposed structure was sensitive to oblique incidence angles after 45°, causing angular dispersion. We noted that the suggested MMA displayed a frequency of 14.1 GHz to create a new absorption band with an incidence angle of 45° in TE mode, and the frequencies 12.8 GHz and 16.2 GHz to create a new absorption band with incidence angles of 35° and 45° at TM mode. The variation in H-field strength components across the metamaterial structure is the primary cause of the difference in magnitude of absorption for the TE and TM modes. As a result, the presented MMA displays outstanding absorption performance with incidence angle insensitivity. The RAB is derived using an equation to evaluate the metamaterial’s absorption capability.

**Figure 5:**
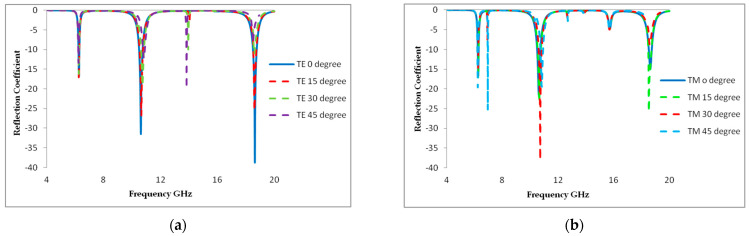
Reflection coefficient results of proposed MA at different incidence angles: (**a**) TE and (**b**) TM.

In Figure 4a,b, the incidence angle varies from 0° to 45°, but the absorption rate is not changed. Thus, this paper also demonstrates incidence angle-insensitive characteristics. Evaluating the absorption rate response in both TM and TE mode allows one to investigate the incidence angle-insensitive properties of the proposed design. The designed metamaterial reflection coefficient is depicted in Figure 5.

### 3.3. Equivalent Circuit Analysis

**Figure 6:**
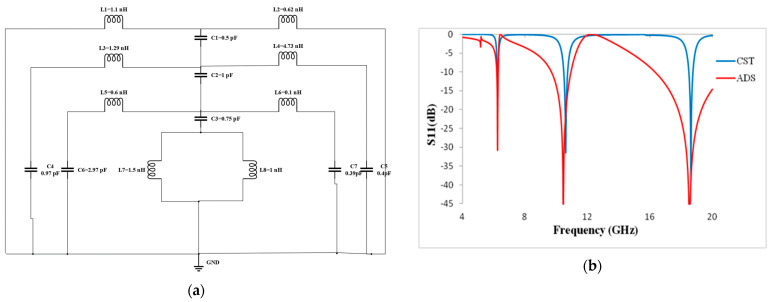
(**a**) Equivalent circuit; (**b**) S_11_ results using ADS and CST simulator.

To describe the electrical behavior of the absorbers, a common strategy is to use the equivalent circuit technique for a metamaterial absorber unit cell. The equivalent circuit of the proposed metamaterial absorber structure is first modeled using ADS (Advanced Design System) software 2021, Carlsbad, CA, USA. Then, the values are manually tuned to obtain the respective resonance frequency that was achieved by the CST, as illustrated in Figure 6a. The split gap is constructed to improve that model’s capacitive impact. The inductor L1 and L2 acts as copper at the bottom. The capacitor C1 acts as a coupling capacitor which is connected between the copper at the bottom and the resonator at the top. The inductors L3 and L4 indicate the outer ring, and the C4 and C5 capacitors indicate the gap. L3 and C4 are connected in parallel, as are L4 and C5. The capacitor (C) is induced by an electric field perpendicular to the component cell’s gap in the equivalent circuit of a unit cell. The coupling capacitor C2 is built in between the outer ring and the inner ring. Inductors L5 and L6 indicate the inner ring, and C6 and C7 indicate the gap. L5 and C6 are connected in parallel, as are L6 and C7. In addition, the coupling capacitor C3 is connected between the inner ring and the H-shaped structure at the center of the patch. The L7 and L8 inductors are connected in parallel, which indicates the H-shaped structure. As a result, the equivalent circuit and CST simulation performed using the proposed unit cell design were able to create three slightly mismatched resonance frequencies. Figure 6b compares the S_11_ results from the equivalent circuit with the CST Microwave Studio software 2019, Aachen, Germany.

### 3.4. Electric Field, Magnetic Field, and Surface Current Distribution

**Figure 7:**
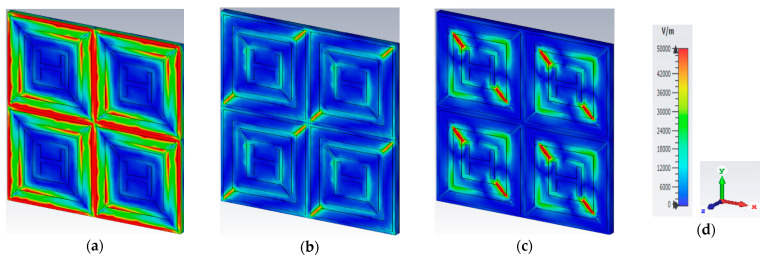
Electric field at (**a**) 6.24 GHz; (**b**) 10.608 GHz; (**c**) 18.624 GHz. (**d**) Scale and axis.

Electric and magnetic field distribution plots are used to evaluate the structure’s physical process. In Figure 7, the electric field distribution at frequencies of 6.24, 10.608, and 18.624 GHz is depicted. When the frequency is 6.24 GHz, the E-field dispersion is highest at the top and bottom positions of the outer square patch resonator structure, indicating that absorption occurs mostly in these areas of the unit cell. The E-field is produced at the outer two split gap resonators for a frequency of 10.608 GHz. The inner ring split gap and four corners of the inner square patch resonator have the highest E-field densities when the frequency is 18.624 GHz.

**Figure 8:**
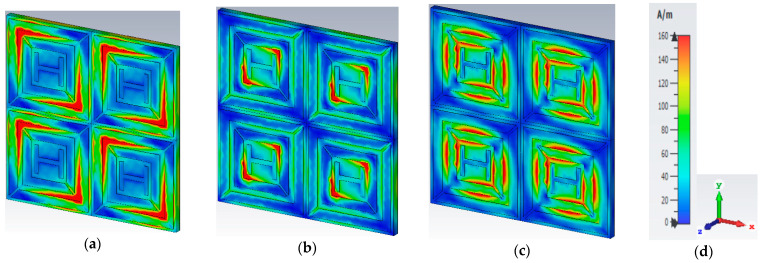
Magnetic field at (**a**) 6.24 GHz; (**b**) 10.608 GHz; (**c**) 18.624 GHz. (**d**) Scale and axis.

Figure 8 displays the magnetic field densities for the triple bands. When the frequency is 6.24 GHz, the H-field dispersion is highest at the outer ring resonator’s left and right positions. The H-field intensity is highest in the inner split ring resonator when the frequency is 10.608 GHz. The inner ring and all four corners of the resonator have the highest H-field at the frequency of 18.624 GHz. The E-field and H-field show opposite excitation at 6.24 GHz, 10.608 GHz, and 18.624 GHz, which satisfies the Maxwell equation requirement. According to the field distribution functions, the fields accumulate along the patch, resulting in the electric resonance.

**Figure 9:**
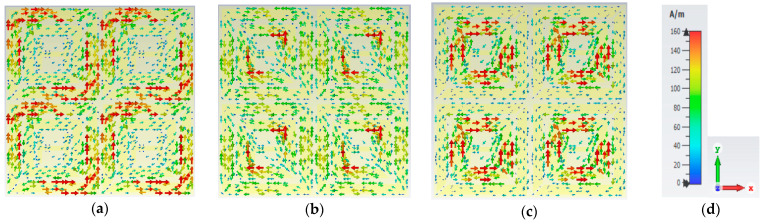
Surface current at (**a**) 6.24 GHz; (**b**) 10.608 GHz; (**c**) 18.624 GHz. (**d**) Scale and axis.

The proposed metamaterial absorber was investigated for its surface current distributions. When interacting with incident EM waves, dipolar responses can be seen where the currents simultaneously flow upwards and downwards. Figure 9 illustrates the surface current situation of the proposed metamaterial absorber at the bottom plane. For the 6.24 GHz and 18.624 GHz resonant frequencies, the currents simultaneously circulate inside and outside of the outer ring and inner ring. At a frequency of 10.608 GHz, the currents are only present at the inner ring’s surface and do not enter the copper lines. The distribution of E-field intensity is located in the opposite direction to that of the surface current density, as seen in Figure 7 and Figure 8. The intensity of the H-field distribution is related to the distribution of the surface current density, as shown in Figure 8 and Figure 9. To put it another way, the H-field has a greater surface current density, and vice versa. Additionally, the current directions of all absorption peaks are anti-parallel to the surfaces of the proposed MA. These anti-parallel current loops create an artificial magnetic dipole moment that is highly linked with the incident magnetic field. As a result, the simultaneous activation of the magnetic resonances is made possible by the suggested MA structure for 6.24 GHz, 10.608 GHz, and 18.624 GHz.

### 3.5. Metamaterial Characteristics

**Figure 10:**
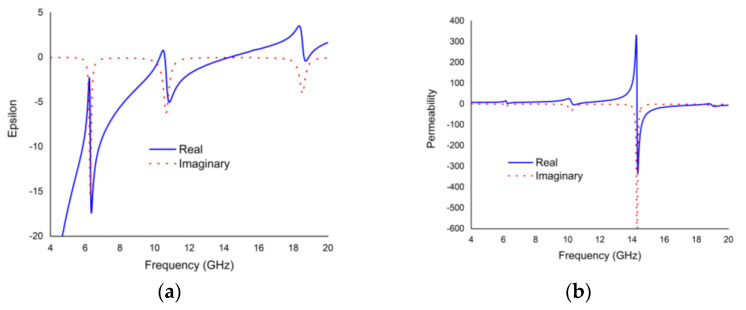
The scenarios of (**a**) permittivity and (**b**) permeability.

The real and imaginary parts of the effective permittivity and permeability of the proposed unit cell are plotted in Figure 10. The proposed metamaterial demonstrates negative permittivity and permeability values for real parts at frequencies of 6.24 GHz, 10.608 GHz, and 18.624 GHz. Thus, the proposed metamaterial exhibits double negative characteristics.

### 3.6. Array Analysis

**Figure 11:**
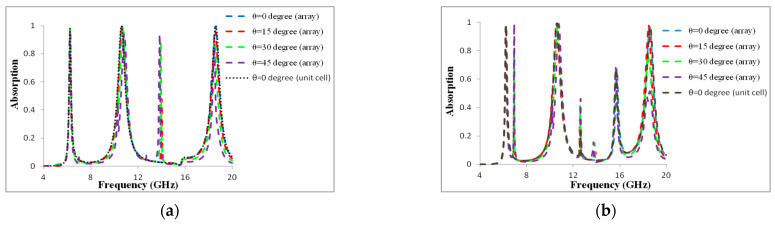
Absorption at (**a**) TE and (**b**) TM mode for a 1 × 2 array and unit cell.

**Figure 12:**
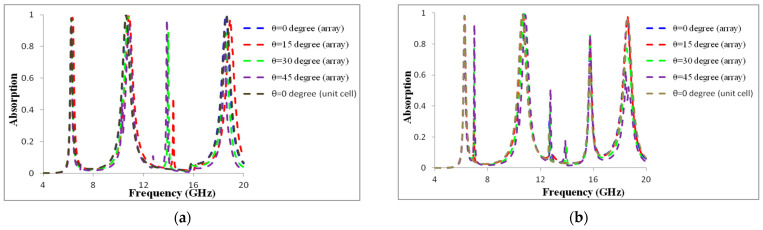
Absorption at (**a**) TE and (**b**) TM mode for a 2 × 2 array and unit cell.

The numerical performance was calculated for initial arrays of 1 × 2 and 2 × 2 sets of the adjacent unit cells in order to demonstrate that was no mutual interaction among neighboring unit cells in a full absorber. Figure 11 and Figure 12 display the absorption performances of these basic arrays for the TE and TM modes. The arrays showed similar findings at normal and oblique incidences, with a slight departure from the resonance frequencies.

The structure’s absorption rates and resonance frequency stayed constant as the angle values changed. The fundamental source of the variance in absorption magnitude for TE and TM modes was determined to be the change in H-field strength components across the metamaterial’s structure. As a consequence, the given MMA exhibited excellent absorption performance while being incident angle-insensitive. We observed the unit cell MMA and the results of both 1 × 2 and 2 × 2 arrays to be almost the same in both TE mode and TM mode.

## 4. Validation of CST Results with HFSS Simulation

**Figure 13:**
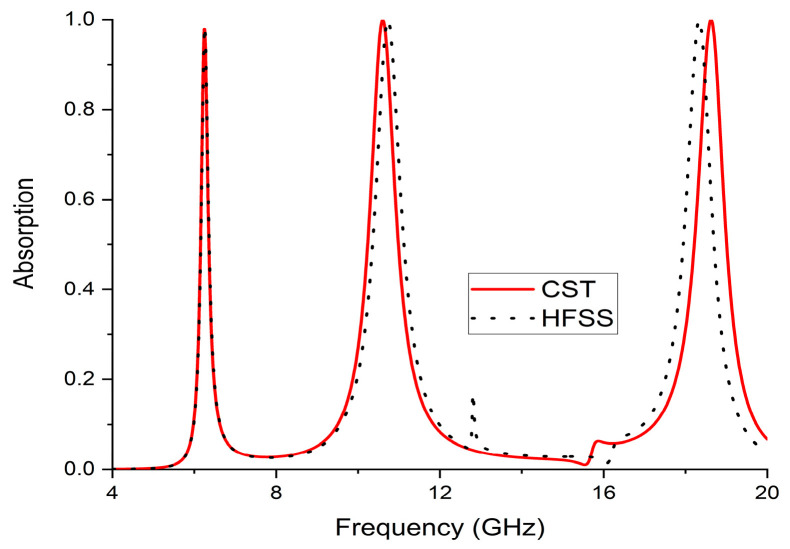
Absorption response using the CST and HFSS simulators.

HFSS was implemented to develop and analyze the proposed unit cell design, which was built using two split rings and an H-shaped structure placed horizontally across a substrate (Rogers RO 4003C) to separate the structure from a continuous copper plane. As shown in Figure 13, we compared the results using the HFSS (High Frequency Electromagnetic Field simulation) simulator and CST Microwave studio. The HFSS simulation findings showed a triple band absorption response at resonance frequencies of 6.25 GHz, 10.77 GHz, and 18.37 GHz, with 97.8%, 99.4%, and 99.7% absorption levels for TE mode. Both simulator findings showed a similar trend, with a slight change because the HFSS simulator utilized the finite element technique and the CST simulator used the finite integration technique.

Table 3 contrasts the results of the suggested MA with those of the previous work on MA based on absorption level, size of the unit cell, design structure, absorption frequency, and other factors. Comparing the suggested MA to previously reported multi-band absorbers, the proposed absorber structure not only reduces the dimensions and thickness of the substrate, but also raises its absorption level. The metamaterial absorbers that have been previously described have multiband properties with better absorption levels due to the use of different types of top resonators, as well as dielectric substrates. The designed MA is more compact and ultra-thin, revealing triple-band absorption rates of 97.8%, 99.9%, and 99.9% at 6.24 GHz, 10.608 GHz, and 18.624 GHz, respectively. This structure also exhibits high Q factors of 62.4, 17.68, and 26.61, as well as RAB values calculated at 32%, 28%, and 21% at all absorption frequencies. Due to the incident angle insensitivity, the designed MA is suited for satellite applications, stealth technology, imaging, and electromagnetic interference reduction.

## 5. Conclusions

We developed a perfect metamaterial absorber with a single metal square patch and a continuous metal ground isolated by a central dielectric substrate that is ultra-thin, triple-band, and incidence angle-independent. The proposed metamaterial absorber exhibits triple-band absorbers. The absorption is exceedingly strong, showing rates of 97.8%, 99.9%, and 99.9% under normal and even oblique (0° to 45°) incident angles, in chosen frequency ranges of 6.24, 10.608, and 18.624 GHz, respectively, for TE mode. TM mode offers the same scenario with slight changes. In order to easily comprehend the modeling and analysis, the effects of the electric field, magnetic field, surface current, and incident angle independency were studied herein. Utilizing ADS Simulator, the equivalent circuit model of the proposed MA was tested. The CST simulation results were also validated by the HFSS simulator. The double negative materials are a unique property of the proposed design, and are widely recognized as metamaterial absorbers which can maintain the thinnest thickness achievable. Due to the superior performance of the proposed MA, this MA could be utilized in radar systems, stealth technology, imaging, and electromagnetic interference reduction.

## Figures and Tables

**Table 1:** The quality factor (Q), FWHM, and RAB results for TE mode.

Frequency (GHz)	FWHM	Q Factor	f_U_; f_L_	RAB
6.24	0.1	62.4	6.3; 6.1	32%
10.608	0.6	17.68	10.7; 10.4	28%
18.624	0.7	26.61	18.7; 18.3	21%

**Table 2:** The quality factor (Q), FWHM, and RAB results for TM mode.

Frequency (GHz)	FWHM	Q Factor	f_U_; f_L_	RAB
6.24	0.1	62.4	6.3; 6.1	32%
10.608	0.6	17.68	10.7; 10.4	28%
18.625	0.6	31.04	18.8; 18.4	21%

**Table 3:** Comparison table of proposed MAs in the previous literature.

Reference	Size (mm)	Frequency Range (GHz)	Design	Absorption (%)	Other Factors
[9]	10.4 × 10.4	3.2, 5.32, 11.15, 16.73	Four-fold symmetry	95.75%,95.93%, 97.69%, 95.64%	Incident angle 0° to 45° and FWHM of 90, 220, 410, and 700 MHz
[17]	8.25 × 8.25	8.85, 14.17	A metallic pentagon patch	90% and 90%	FWHM 57.3%
[21]	11 × 11	7.8–14.7	Quadrangular frustum pyramids	Above 90%	RAB 61.6% and polarization-independent
[28]	34.036 × 34.036	2.7, 3.26, 4.05	G-shape resonator	60%, 91.5%, 70.3%	Q-factor 271 at 3.26 GHz
[39]	10 × 10	3.68, 8.58, 10.17, 14.93	Conductive cross dipoles	96.15%, 99.1%, 99.75%, 98.75%	Polarization-insensitive (0°, 15°, 30°, 45°, 60°)
Proposed design	8 × 8	6.24(C-band), 10.608(X-band), 18.624(K-band)	Two metallic square patches with H-shaped structure	97.8%, 99.9%, 99.9%	Incident angle-insensitive (0°, 15°, 30°, 45°); Q-factors of 62.4, 17.68, 26.61; and RAB calculated as 32%, 28%, and 21%

## Data Availability

All the data are available within the manuscript.
